# They Don’t Care About Us! Care Personnel’s Perspectives on Ambient Assisted Living Technology Usage: Scenario-Based Survey Study

**DOI:** 10.2196/10424

**Published:** 2018-09-24

**Authors:** Julia Offermann-van Heek, Martina Ziefle

**Affiliations:** 1 Human-Computer Interaction Center RWTH Aachen University Aachen Germany

**Keywords:** Ambient Assisted Living technologies, assistive technologies, care professionals, diverse care contexts, web-based survey, quantitative scenario-based approach, technology acceptance, user diversity

## Abstract

**Background:**

Demographic change represents enormous burdens for the care sectors, resulting in high proportions of (older) people in need of care and a lack of care staff. Ambient Assisted Living (AAL) technologies have the potential to support the bottlenecks in care supply but are not yet in widespread use in professional care contexts.

**Objective:**

The objective of our study was to investigate professional caregivers’ AAL technology acceptance and their perception regarding specific technologies, data handling, perceived benefits, and barriers. In particular, this study focuses on the perspectives on AAL technologies differing between care professionals working in diverse care contexts to examine the extent to which the care context influences the acceptance of assistive technologies.

**Methods:**

A Web-based survey (N=170) was carried out focusing on professional caregivers including medical, geriatric, and disabled people’s caregivers. Based on a scenario, the participants were asked for their perceptions concerning specific technologies, specific types of gathered data, and potential benefits of and barriers to AAL technology usage.

**Results:**

The care context significantly impacted the evaluations of AAL technologies (F_14,220_=2.514; *P*=.002). Professional caregivers of disabled people had a significantly more critical attitude toward AAL technologies than medical and geriatric caregivers, indicated (1) by being the only caregiver group that rejected evaluations of AAL technology acceptance (F_2,118_=4.570; *P*=.01) and specific technologies (F_2,118_=11.727; *P*<.001) applied for gathering data and (2) by the comparatively lowest agreements referring to the evaluations of data types (F_2,118_=4.073, *P*=.02) that are allowed to be gathered.

**Conclusions:**

AAL technology acceptance is critical because of technology implementation reasons, especially in the care of people with disabilities. AAL technologies in care contexts have to be tailored to care professional’s needs and concerns (“care about us”). The results contribute to a broader understanding of professional caregivers’ needs referring to specific data and technology configurations and enclose major differences concerning diverse care contexts. Integrating these findings into user group-tailored technology concepts and communication strategies will support a sustainable adoption of AAL systems in professional care contexts.

## Introduction

### Background

Demographic change involves higher proportions of older people and people in need of care, bringing the care sector to its knees due to personnel, economic, and organizational shortcomings [1,2]. Geriatric care, nursing care, and—as a comparatively new development [3]—care institutions for (older) disabled people suffer badly from a lack of care personnel in combination with raising needs of care for older (geriatric care), chronically ill (curative care), and disabled (care of the disabled) people [4,5].

The development of technical innovations is proceeding constantly to relieve care staff, complement care supply, enhance safety in emergencies, and enable a largely autonomous life for people in need of care [6]. Within these developments, diverse Ambient Assisted Living (AAL) technologies and systems [7,8] enable monitoring of vital parameters, detecting falls and emergencies, and a longer stay at home using smart home technologies [9,10].

Such technologies and systems are rarely used in both real-life and professional working environments [11]. Beyond availability and technical possibilities, users’ acceptance and the broad willingness to use these systems are decisive for a sustainable integration of AAL technologies in everyday life [12]. As recently reported [13], there are considerable differences in AAL acceptance between people in need of care and professional caregivers, indicating a more critical and restraint attitude of care staff compared with people in need of care and their relatives.

The professional caregivers’ perspectives on specific AAL technologies and on the data gathered in line with perceptions regarding benefits of and barriers to such systems are not known. This study, therefore, takes professional caregivers’ perspectives on AAL technologies into account, comparing different care contexts—geriatric care, medical care, and disabled people’s care.

In the following sections, we have presented the theoretical background starting with examples of current AAL technologies and systems, followed by AAL acceptance studies in professional care contexts.

#### Ambient Assisted Living Technologies and Systems

Assisting technologies or systems contribute to an increased autonomy in everyday life and are applied in care for prevention and rehabilitation, summarized under the term “Ambient Assisted Living” technologies. They cover diverse functions such as monitoring, detection, or reminders [10,14,15] and have the potential to empower collaborations in self-care [16].

Integrating Information and Communication Technologies (ICT; eg, cameras, microphones, motion sensors) into people’s living environments enables monitoring to enhance safety by detecting falls and emergencies in private [17] as well as professional care environments, for example, care institutions, hospitals, or retirement homes [18]. In addition, some approaches aim for monitoring and tracking outdoors using radio frequency identification [19], for example, to locate missing people suffering from dementia or confusion.

In addition to safety-related goals, automated technologies are used to facilitate everyday life (eg, memory aids, home automation) [20,21]. Enabling communication with families, friends, doctors, and caregivers by integrating ICT into home environments is a further aim of AAL [10]. Besides technologies integrated into devices and rooms, wearable technologies (eg, emergency arm strap) worn on the body or integrated into clothes present a further field of AAL enabling communication with smart home environments [7,22]. Although a considerable number of systems are already available on the market, success and sustainable integration of those systems have failed to appear so far [11,23]. Thus, reasons for their failure have to be investigated as caregivers’ acceptance of assistive technologies is of paramount importance for successful integration and usage of AAL technologies; as relevant stakeholders and users of these systems, professional caregivers’ perceptions, needs, and willingness to adopt AAL technologies need to be focused upon.

#### Acceptance of Ambient Assisted Living Technologies in Professional Care Contexts

Overall, AAL technologies were mostly evaluated positively; key drivers to use AAL technologies are the benefits of independent and autonomous living as well as a longer stay at the own home for older, chronically ill, or disabled people [13,24]. At the same time, feelings of isolation [13,25] and surveillance as well as perceived threat of privacy violations [26,27] were key barriers that impeded the integration of AAL technologies into people’s living environment.

The perspectives and perceptions of care professionals on integrating AAL technologies into their working environments have rarely been considered in acceptance research so far. Frequently, the research investigates care in emergency or ambulance contexts involving perspectives of (elderly) patients and care professionals [28,29]. One study has considered caregivers and their perceptions toward in-home monitoring technologies [30] and one has derived guidelines for design and implementation in the context of professional care environments [31]. Overall, a positive attitude of nursing staff toward health care information technology has been revealed, while poor system design and fear of dehumanizing patient care have been reported to be the main barriers of health care information technology usage [32]. Furthermore, ICT support in dementia care [33] has showed a positive general perception of ICT, but similarly diverse and mixed evaluations during technology implementation. In contrast to those—predominantly—positive generic attitudes toward technology usage in care contexts, a recent, more specific study revealed quite critical and restraint attitudes of professional nursing staff toward AAL technologies compared with more positive perspectives of disabled participants, the relatives of disabled persons, and “not”-experienced participants (persons without experiences with care) [13].

These diverse and partly contradicting results in different care contexts might serve as a starting point for explaining why AAL technologies are not widely used in professional care contexts yet. In addition, the thin body of knowledge in this context stresses the necessity for more specific research exploring possible reasons for accepting or declining care technology, such as the type of AAL technology, the issue of data collection and privacy handling, as well as the impact of different care contexts on AAL technology acceptance [34].

As a theoretical base, the acceptance of assisting ICT has been grounded by long-time established acceptance models such as the Technology Acceptance Model (TAM) [35] and the Unified Theory of Acceptance and Use of Technology (UTAUT) [36], which had been developed for ICT usage mostly in healthy persons in the working context. For the specific nursing and care requirements, those models of technology acceptance are not sufficient, mostly because the main determinants of acceptance models—ease of using a system and perceived usefulness—might be an oversimplification of the situation in complex care settings, where not only the technology but also the fragile situation of patients, in line with the sensitive relation between caretakers and caregivers, is of importance. Furthermore, previous acceptance models do not consider different caring contexts and the inherent trade-offs between simultaneously existing positive and negative usage motives [37].

### Objective and Aim of the Study

Due to abovementioned reasons, it was necessary to use a qualitative approach first; interviews were conducted with professional caregivers working in diverse care areas (n=6) to identify challenges in care and the perceived benefits of as well as barriers to AAL technology usage from a care staff’s perspective. The differentiated look on professional caregivers allowed us to investigate what assistive technology should and should not do. Based on the qualitative results of this preceding study, the Web-based questionnaire for this study was conceptualized.

Therefore, this study aimed to quantitatively investigate the professional caregivers’ acceptance of assistive technologies in professional care contexts, differentiating between geriatric care, medical care, and disabled people’s care. This investigation was driven by the following research questions:

1. Do professionals of different care contexts differ with respect to their perceptions of AAL technologies?
2. Do professionals of different care contexts differ with respect to their willingness to share care-related data?
3. Do professionals of different care contexts differ with respect to their willingness to be assisted by specific AAL technologies in their daily routines?
4. On a data level, which are the main predictor variables for the AAL acceptance across the different care contexts?

## Methods

### Methodology

In order to reach a larger sample of care professionals, a Web-based survey was developed and specifically tailored to professional caregivers working in diverse care contexts. A preceding interview study focused on professional caregivers’ daily routines, their perceptions of different assistive technologies, and their wishes and needs. These qualitative results enabled the development of a scenario- and Web-based survey that addresses professional caregivers in a realistic and comprehensible way.

### Research Variables

As an independent variable, we explored the care context contrasting 3 areas: (1) geriatric care; (2) nursing care; and (3) disabled people’s care and support. Naturally, these are not distinct categories as sometimes, there are overlaps across these care areas in real-life care settings. However, the responding caregivers had to assess themselves in terms of their main professional area. Thus, we took their self-assessment as an expert classification.

As dependent variables, we analyzed different acceptance ratings. First, participants answered items with respect to AAL technology acceptance, differentiating among data storage, access, and collection, as well as perceived benefits and barriers respecting AAL technologies. The items for these areas were taken from a preceding qualitative interview study with professional caregivers of different care areas. Furthermore, the next dependent variable relates to different types of gathered data and different types of technologies used for AAL assistance. All constructs, the respective items, and their evaluations have been presented in Multimedia Appendix 1.

### Empirical Design of Web-Based Survey

Overall, the survey contained 14 questions pictured on 9 pages (including the starting page, final page, and scenario introduction). Measuring was operationalized by 6 forced-choice questions, open comment fields, and 82 items departed in 8 thematic blocks. These items were block wise randomized and had to be evaluated on 6-point Likert scales (1=min: “I strongly disagree” to 6=max: “I strongly agree”). Thereby, values <3.5 indicated rejection, whereas values >3.5 indicated approval. During the Web-based survey, participants had the opportunity to review and change their answers if desired.

Demographic characteristics (age, gender, education, and duration of professional experience) and the care sector of respondents (ie, geriatric care, nursing care, and disabled people’s care and support) represented the questionnaire’s first part of the survey. In the second part, participants’ attitudes toward technical self-efficacy (4 items, alpha=.884; based on [38]), their needs for privacy (6 items, alpha=.833; based on [39,40]), and their interpersonal trust (3 items, alpha=.793; based on [41]) were assessed.

To ensure that all participants refer to the same baseline concerning the evaluation of AAL technology, a scenario approach was adopted; the participants should imagine the integration of an AAL system into their professional working environment. Room sensors, microphones, video cameras, and ultrasonic sensors were introduced as part of the AAL system, and their functions within the AAL system were detailed (eg, automatic opening and closing of doors and windows, reminders, and alarms [emergencies, falls]).

Subsequently, participants were asked to evaluate perceived potential benefits (14 items, alpha=.923) and potential barriers (17 items; alpha=.861) referring to the described AAL system. Both benefits and barriers of the described AAL system were obtained from the interview study. Furthermore, participants indicated whether they would accept gathering different types of data (14 items, alpha=.856; based on the information needed to realize technical functions). Afterward, participants assessed different technologies to gather data (using 12 items, alpha=.892; based on technical configurations of the AAL system). Additionally, participants assessed data access (alpha=.802) and data storage (alpha=.760) issues, each using 3 items referring to diverse types of data (video data, audio data, position data, and room data).

The acceptance of AAL system was evaluated using 6 statements (alpha=.932; eg, “I find the described AAL system useful”). Finally, participants could reason their opinions and their feedback concerning the study on an optional basis.

Before the study was started, the Web-based survey was pretested by communication scientists concerning comprehensibility and technical functionality. Additionally, pretests with “laypeople” were conducted to ensure comprehensibility and to enable the estimation of the length of time participants would need to fulfill the survey.

#### Recruitment and Sample

As the study aimed at reaching professional caregivers exclusively, it was not a typical convenience sample. The link to the e-survey was purposefully distributed (1) in specific Web-based networks (geriatric care and nursing care); (2) via mail by personal contact to caregivers (mostly geriatric and nursing care); and (3) via mail by project contact to care institutions (care of people with disabilities). Participation in the open survey was completely voluntary, and no monetary incentives were offered.

Of course, the collection of participants is one of the most important issues in empirical studies. In this case, it was especially sensitive as professional caregivers were asked to unveil possibly sensitive data and share personal insights into their working environment. Prior to data collection, we intensively discussed aspects concerning data protection and privacy policy with a German umbrella organization of care personnel and the main organization for people with disabilities and decided to organize data collection about their network without asking people to unveil their specific institution.

For this study, we did not seek ethical approval from the ethics committee as our study falls in the category where no such approval is necessary in Germany. This category spans all noninvasive, nonclinical research on human subjects, where subjects are transparently informed about the purpose, aim, and risks of the studies and when these risks are reasonably low. Prior to starting the procedure, participants were informed that it is of high importance to understand free opinions and attitudes on assistive technologies from the caregivers’ (expert) perspective and that we would be delighted if they would share their opinions with us. In addition, we informed participants about the duration of the survey, the main purpose, and our department as investigators. Furthermore, we ensured a high standard of privacy protection and let participants know that none of their answers could be traced back to them as persons. Demographic data were also submitted voluntarily, and all participants were informed that their personal data would be deleted from our encrypted hard drives on request. After these careful explanations, participants reported feeling well informed about the purpose and aim of this study and about their freedom to quit participation at any time. Regarding the privacy policy explanations, participants reported understanding that high standards were applied, and they deliberately accepted participation. From comments in the open question fields at the end of the survey, we learned that participants were interested in the topic and were keen to look at the results, which we assured them to receive.

For completing the questionnaire, participants took on average 20 minutes, and data were collected in Germany from April to June, 2017. Overall, 287 participants opened the Web-based survey and 4.9% (14/287) participants canceled the survey after viewing the introducing start page. Thus, 95.1% (273/287) of the respondents participated in the survey; 64.8% (186/287) participants filled out the survey completely. From these participants, 16 were excluded from further analyses because they did not match the criterion of being a professional caregiver within the areas of geriatric, nursing, and people with disabilities’ care (eg, employees of administration). Finally, 59.2% (170/287) care professionals were considered for the data analysis.

The mean age of participants was 36.26 (SD 11.23) years, with a higher proportion of female (74.7%, 127/170) care professionals; 42.2% (72/170) participants indicated a completed apprenticeship as the highest educational level, whereas 23.0% (39/170) reported holding a university degree or a university entrance diploma. Furthermore, 7.6% (13/170) indicated holding a secondary school certificate, and 4.2% (7/170) reported holding other certificates.

All participants were experienced care professionals; 25.3% (43/170) participants reported working in geriatric care, 22.9% (39/170) in medical care, and 51.8% (88/170) in disabled people’s care. On average, care professionals had long-term experiences, with 42.8% (73/170) of them having >10-year experience and 42.8% (73/170) having between 3- and 10-year professional experience; 14.4% (25/170) reported having <3-year professional experience.

Regarding attitudinal aspects, participants had a medium technical self-efficacy (mean 3.4 [SD 0.7]; min=1, max=6) and a middle interpersonal trust (mean 3.5 [SD 0.8]; min=1, max=6). Participants’ needs for privacy and data security were on a moderate positive level (mean 4.2 [SD 0.9]; min=1, max=6).

#### Data Preparation and Analysis

For data analysis, only completely filled datasets and only participants with a professional care background were considered. As additional adjustment criterion, datasets with an atypical timestamp were excluded, indicated by a processing time <50% of the calculated median referred to all completed datasets’ processing time (18 minutes). Regarding Internet Protocol (IP) address check, the link to the survey used for direct invitations via mail and used on social Web-based networks was related with the condition that one IP address was allowed to access the Web-based survey only once. For the link to the survey distributed via project contact to care institutions for people with disabilities, using this condition was not possible. In these institutions, the caregivers filled the survey at stationary computers, and we had to allow using the same IP address multiple times. As this institution for disabled people’s care is part of the research project, there is a proprietary interest regarding the study’s results.

Before descriptive and inference analyses were performed, item analyses were calculated to ensure measurement quality. A Cronbach alpha >.7 indicated a satisfying internal consistency across the scales. Data were analyzed descriptively, as well as by linear regression analyses and, with respect to effects of the professionals’ care context and user diversity, by multivariate inference analyses (significance level was set at 5%). Furthermore, post hoc tests were analyzed using Tukey honestly significant difference test.

## Results

### Fundamental Differences in Ambient Assisted Living Technology Perception

**Figure 1:**
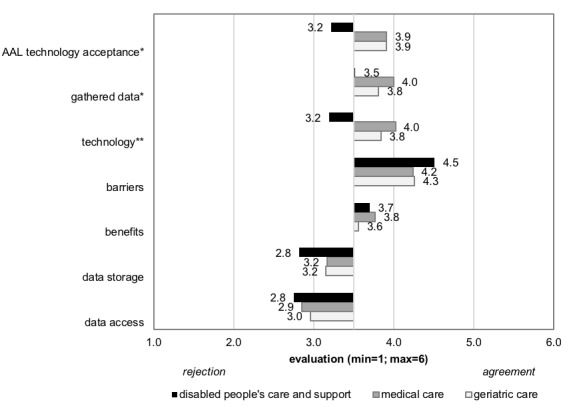
Results for the constructs of Ambient Assisted Living (AAL) technology perception (*P<.05; **P<.01).

We have reported descriptive findings as well as inference statistics differentiating between care professionals working in different care contexts (group differences are reported based on post hoc tests [Tukey honestly significant difference]). Looking at the results for the constructs of AAL technology perception (Figure 1), significant differences for the 3 care contexts were revealed (*F*_14,220_=2.514, *P=*.002). Multimedia Appendix 1 presents means and SDs of all items for the whole sample and the 3 care contexts.

Participants working in the area of disabled people’s care indicated a significantly lower *acceptance of AAL technologies* (*F*_2,118_=4.570; *P*=.01) than those working in geriatric and medical care. Furthermore, regarding the *data that are allowed to be gathered,* the perception was significantly different (*F*_2,118_=4.073; *P*=.02); participants working not only in medical care but also in geriatric care showed more positive evaluations compared with participants working in disabled people’s care. Regarding *technologies that can be used to gather data*, the same result was found (*F*_2,118_=11.727; *P<*.001); participants working in medical and geriatric care differed significantly from those working in disabled people’s care, who indicated a more negative attitude toward specific technologies. In contrast, potential *benefits* of (*F*_2,118_=0.350; *P*=.71) and *barriers* to (*F*_2,118_=1.853; *P*=.16) AAL technology usage were not found to be significantly different across the care contexts (disabled people’s care: mean 4.5 [SD 0.7]; medical care: mean 4.2 [SD 0.7]; and geriatric care: mean 4.3 [SD 0.9]). Issues of d*ata access* (*F*_2,118_=.340; *P*=.71) and *data storage* (*F*_2,118_=2.235; *P*=.11) were not found to be significantly different as well, showing a homogenous evaluation independent of the care context.

### Specific Differences in Data, Technology, and Acceptance Evaluations

In this section, we present the significant differences within the evaluation of AAL technology acceptance, applied technologies, and data collection in more detail.

#### Willingness to Share Care-Related Data

**Figure 2:**
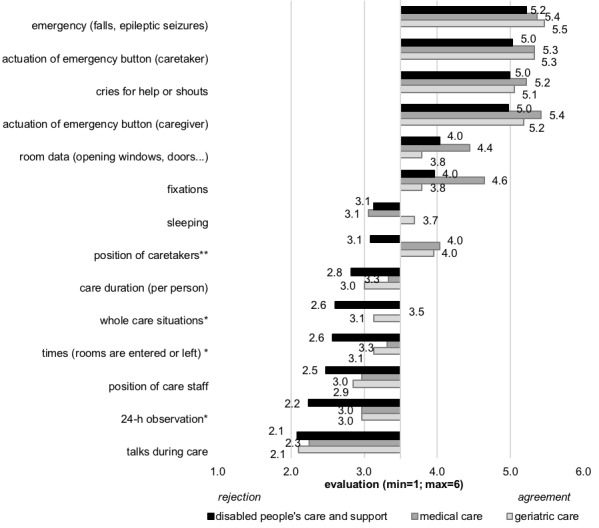
Results of different types of potential gathered data (*P<.05; **P<.01).

Participants evaluated their willingness to share 14 different types of data that could be usefully collected for AAL technology usage. The evaluation of *gathered data* strongly depended on care context. Figure 2 shows the results for all types of data, and Multimedia Appendix 1 presents all means and SDs. For data collected in the context of *emergencies* (eg, *actuation of emergency buttons* [*caretakers; F*_2,151_=1.729; *P*=.18], *cries for help or shouts* [*F*_2,151_=.536; *P*=.59]), the evaluation was positive and approved by all caregivers regardless of the care context. The gathering of data concerning *rooms* (*opening windows* and *doors* [*F*_2,151_=1.709; *P*=.19]) and *fixations* (*F*_2,151_=2.891; *P*=.06) did not significantly differ with regard to the care context, even though the highest evaluations were given by the group of medical caregivers. A slight but not significant difference was revealed for data regarding *sleeping* (*F*_2,151_=2.315; *P*=.10), which was slightly rejected by medical care (mean 3.1 [SD1.1]) and disabled people’s care (mean 3.1 [SD 1.5]) professionals, while slightly accepted by geriatric care professionals (mean 3.7 [SD 1.7]).

The most striking and significant difference was present for data collection about *the position of caretakers* (*F*_2,151_=8.283; *P*<.001), which was moderately accepted to be collected by participants working in medical (mean 4.0 [SD 1.1]) and geriatric (mean 4.0 [SD 1.6]) care, while rather rejected by participants working in disabled people’s care (mean 3.1 [SD 1.4]).

Collecting data about *care duration (per person; F*_2,151_=1.351; *P*=.26) was rejected by all participants. The collection of data about *whole care situations* (*F*_2,151_=4.517; *P*=.01) and *times (rooms are entered or left*; *F*_2,151_=4.049; *P*=.02) was generally rejected by all participants, but it differed significantly across care contexts; people working in disabled people’s care showed a stronger rejection than medical care and geriatric care professionals.

Descriptively, a similar result was found for data about *positions of caregivers*, indicating a higher rejection by people working in disabled people’s care, even though the differences missed statistical significance (*F*_2,151_=1.609; *P*=.20). There was a significantly higher rejection of data concerning a *24-h observation* (*F*_2,151_=4.080; *P*=.02) by disabled people’s care professionals than by medical and geriatric care professionals. Finally, the gathering of data about *conversations during care* (*F*_2,151_=.199; *P*=.82) was rejected most strongly by all participants regardless of the care context.

#### Willingness to be Assisted by Specific Ambient Assisted Living Technologies in Daily Routines

**Figure 3:**
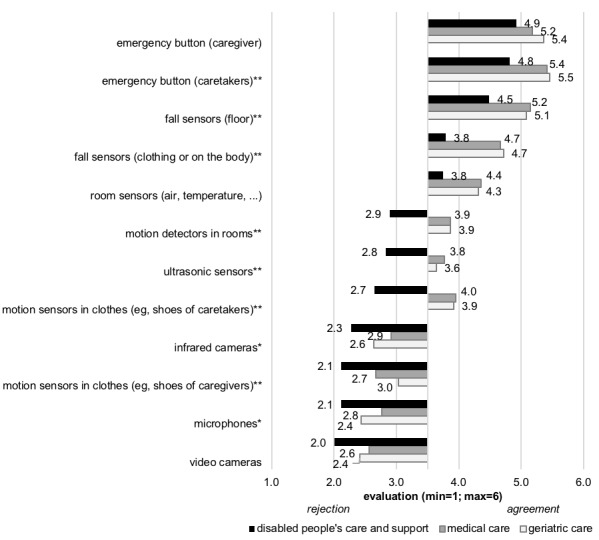
Results of different types of potential applied technologies (*P<.05; **P<.01).

Overall, 12 different types of AAL technologies were evaluated, and the outcomes are depicted in Figure 3. Again, Multimedia Appendix 1 presents all means and SDs for the whole group of participants as well as the 3 care contexts. First, the usage of *emergency buttons* was found to be most positive (*caregivers: F*_2,151_=2.281; *P*=.11; *caretakers*: *F*_2,151_=6.362; *P*=.002). Medical care and geriatric care professionals showed higher evaluations concerning *emergency buttons* that are activated by *caretakers* than the evaluations of disabled people’s care professionals.

The use of *fall sensors integrated into the floor* (*F*_2,151_=4.962; *P*=.008) was also rated significantly more positively by medical and geriatric caregivers than by people working in disabled people’s care. *Fall sensors in clothes or on the body* were evaluated less positively than fall sensors on the floor, but again, (*F*_2,151_=7.908; *P*=.001) disabled people’s care professionals (mean 3.8 [SD 1.6]) showed less positive assessments compared with geriatric (mean 4.7 [SD=1.3]) and medical (mean 4.7 [SD 1.2]) care professionals. A similar evaluation pattern occurred for *room sensors*, even though statistical significance was not reached (*F*_2,151_=2.752; *P*=.07).

*Motion detectors in rooms* (*F*_2,151_=8.494; *P*<.001), *ultrasonic sensors* (*F*_2,151_=7.315; *P*=.001), and *motion detectors in the clothes of caretakers* (*F*_2,151_=15.271; *P*<.001) were all evaluated slightly positively by medical and geriatric care staff. However, they were rejected by disabled people’s care professionals.

**Table 1:** Final regression model for care staff in 3 different care contexts.

Group of participants and dimension		B^a^	Standard error B	beta	t^b^	Variance inflation factor	Adjusted r^2c^
**Geriatric care**							.839
	Technology	.961	.126	.658	7.605	1.389
	Barriers	−.421	.124	−.253	−3.400	1.397
	Benefits	.424	.125	.295	3.387	1.017
**Medical care**							.604
	Technology	.742	.162	.558	4.573	1.164
	Benefits	.450	.140	.392	3.215	1.164
**Disabled people’s care**							.621
	Technology	.581	.156	.388	3.726	1.664
	Benefits	.434	.118	.340	3.687	1.347
	Data	.477	.151	.291	3.156	1.293

^a^B: regression coefficient B.

^b^t: t-statistic (coefficient divided by its standard error).

^c^r^2^: coefficient of determination.

In contrast, the usage of *infrared cameras* (*F*_2,151_=8.494; *P*=.048), *motion detectors in the clothes of care staff* (*F*_2,151_=8.494; *P*=.004), *microphones* (*F*_2,151_=8.494; *P*=.046), and *video cameras* (*F*_2,151_=8.494; *P*=.05) was rejected by all participants, even though the most negative evaluation was prevailing in disabled people’s care professionals.

#### Predictors for the Acceptance of Ambient Assisted Living Technology

Finally, to analyze whether different factors were relevant for AAL technology acceptance in different care contexts, regression analyses were performed. Table 1 shows the linear regression models. The sum-score AAL technology acceptance was integrated as a dependent variable, whereas the sum-scores of perceived benefits, perceived barriers, types of gathered data, the specific technology types, data access, and data storage were integrated as independent variables within the linear stepwise regression analysis.

The final regression model for geriatric care professionals explained 83.9% variance in AAL technology acceptance, grounded on the type of technology, in particular, and on perceived barriers and perceived benefits. In comparison, the final regression model for medical care staff explained 60.4% variance in AAL technology acceptance based on two dimensions—the applied technology and perceived benefits. In contrast, the final regression model for disabled people’s care professionals explained 62.1% variance in AAL technology acceptance and was influenced by the applied technology, perceived benefits, and the types of gathered data.

## Discussion

### Acceptance of Ambient Assisted Living Systems

In contrast to previous research results reporting mostly positive evaluations of ICT and assistive technologies in care [24,32,33], professional care staff has reportedly been more critical concerning the integration of AAL technologies into their professional routine [13,34].

The evaluations of which data can be gathered and which specific technologies should be used revealed yet underexplored insights into the perceptions of care professionals; the only accepted data collection was regarding emergencies, whereas the collection of other data types was, at the utmost, tolerated if not rejected. The negative assessment had been confirmed by open comments in the questionnaire and was also voiced in the preceding interviews. Apparently, care staff evaluations contradict the reasons why AAL systems can be useful at all: those systems can only be efficiently used if data about the patient and his or her location, health status, and care situation are recorded and, if necessary, interpreted by remote medical services. The evaluation of specific technologies showed similar findings; participants indicated to only accept quite static technologies (eg, emergency buttons), which record static, binary data (eg, door open or closed). In line with previous research [42], more complex AAL technologies (eg, cameras, microphones, and life-logging) were—owing to their still higher potential of violation of privacy—broadly rejected in the care context. Participants’ feedback suggests that the major concern is regarding the sneaking suspicion that the collected data will be not only stored for long term but also accessible to others. Here, a general distrust toward illegal data access and abuse by third parties becomes obvious.

The negative attitude might also be attributed to the applied scenario-based approach. Previous research has shown that the methodology used to capture acceptance reactions modulates outcomes considerably; acceptance after hands-on experience with AAL technologies has been much more positive [26] compared with assessing the acceptance through scenario analyses, in which participants should envision the usage of assistive home technologies. Possibly, professional care staff would have evaluated AAL technologies more positively if they had the chance to test these technologies in their everyday professional life and rely their perceptions on own experiences.

### Diversity of Care Matters

In addition to personal characteristics, for example, experience with technology, which is known to impact AAL technology acceptance [32,34], the interview findings revealed that the working conditions in care context are decisive for AAL technology acceptance in the sensible field of care. This study confirms the influence of the care context. While medical and geriatric care professionals are generally more positive toward AAL technologies, the professionals working in disabled people’s care are more reluctant toward the usefulness of AAL systems and perceive higher concerns regarding data collection in the care situation.

We assume that the differences especially in evaluations of data and technology configurations are caused by disparate internal perspectives of the care institutions. Geriatric and especially medical care are concentrated on the short-term and temporary care of old and chronically ill patients and, therefore, focus on patients’ safety as well as substantial improvement in health. Additionally, geriatric and medical caregivers are involved in high numbers of emergencies, in which monitoring technologies are widely used. On those grounds, geriatric and medical caregivers might have a more positive attitude toward assistive technologies. In contrast, care institutions for disabled people have a completely different disputation. They represent a long-term stationary home and, besides safety issues, focus especially on the protection of human dignity, rights, and privacy of their residents. Therefore, caregivers of disabled people probably have a more restraint attitude toward assistive technology and are specifically critical toward the collection of personal data.

Overall, care does not equal care; the diversity of care needs to be considered in the development process of assistive technologies and especially in the way those technologies are introduced and implemented in daily care routines of care institutions.

### Limitations and Further Research

There are some limitations to be considered in future research. While we revealed a basically negative attitude toward (data collection in) AAL systems, stakeholder-specific reasons underlying participants’ reluctance are not known. Possible reasons against using AAL systems might include concerns that (1) employers could control the quality of care (staff); (2) responsibility claims could be pleaded by family members (staff); (3) a low usability of technology might overcharge the technology competence (staff); (4) personal data could appear in public (patient); (5) emergency help would contradict life-end decisions (patient); and (6) a lower supply quality by insurances (family members) or extra financial burden due to costly technology (family members). Future research should clarify which of these reasons should be addressed by adequate information and communication strategies.

A further limitation is related to the applied method and sample issues. Due to our scenario-based approach, the evaluations based on a fictional, and not real, AAL system could have led to an overestimation of potential barriers especially fears concerning data security [26] as well as a general discomfort of being monitored in intimate (care) situations [42]. We, therefore, aim for hands-on evaluations of AAL technologies in diverse professional care environments (ie, in institutions for geriatric, medical, or disabled people’s care).

Moreover, there are sample-related aspects to be considered. Most of our participants were women. Even though this is consistent with higher proportions of women working in care institutions [43], research should aim at exploring more male caregivers to analyze whether acceptance positions are impacted by gender roles.

Furthermore, as we only included participants from one country, outcomes are limited to the German health care system and perspectives on AAL. Future research should extend the perspectives to enable a direct comparison of AAL acceptance as well as data and technology perceptions in different countries and cultures [37].

### Application Potential of the Findings

Findings can be used for the development, design, and configuration of AAL technologies as well as for health care and nursing management issues. As data are not needed to be stored for a long-term (only direct processing) and can be processed by the system for nearly all functions, targeted communication strategies could inform the handling of data (eg, only processing not storage). The transparency and the honesty of communication strategies are essential to inform caregivers about the usefulness of AAL systems for them (support in care routine), for the institution (efficiency), and also for patients and family members (patient safety). In addition, the policy of an institution regarding how data are handled should be explicitly made. Likewise, communication strategies could be tailored to diverse care contexts and their particularities in a more detailed and satisfying way. This is especially important as the feedback from our participants during the preceding interview study and also in comment fields during the Web-based survey (“they don’t care about us”) showed that care personnel often do not feel their needs to be heard and appreciated by the care institution’s management, policy, and society. If care personnel are considered as a valuable part in the process of integration of assistive technologies, all stakeholders—caregivers, caretakers, and patients—will benefit alike.

## Appendix

Multimedia Appendix 1 Descriptive statistics (means and SDs) for the whole sample and the 3 care contexts.

## Abbreviations

AAL: Ambient Assisted Living
ICT: Information and Communication Technologies
IP: Internet Protocol

## References

[1] Pickard L (2013). A growing care gap? The supply of unpaid care for older people by their adult children in England to 2032. Ageing and Society.

[2] Walker A, Maltby T (2012). Active ageing: A strategic policy solution to demographic ageing in the European Union. Int J Social Welfare.

[3] Poore C (2007). Disability in twentieth-century German culture. Disability in Twentieth-Century German Culture.

[4] Shaw JE, Sicree RA, Zimmet PZ (2010). Global estimates of the prevalence of diabetes for 2010 and 2030. Diabetes Research and Clinical Practice.

[5] Harper S (2014). Economic and social implications of aging societies. Science.

[6] Wiles JL, Leibing A, Guberman N, Reeve J, Allen RES (2012). The meaning of “aging in place” to older people. Gerontologist.

[7] Memon M, Wagner SR, Pedersen CF, Beevi FHA, Hansen FO (2014). Ambient Assisted Living Healthcare Frameworks, Platforms, Standards, and Quality Attributes. Sensors.

[8] Frank S, Labonnote N (2015). Monitoring technologies for buildings equipped with ambient assisted living: Current status and where next.

[9] Juan C, Xiang C, Minfen S (2013). A framework for daily activity monitoring and fall detection based on surface electromyography and accelerometer signals. IEEE J Biomed Health Inform.

[10] Kleinberger T, Becker M, Ras E, Holzinger A, Müller P, Stephanidis C (2007). Ambient intelligence in assisted livingnable elderly people to handle future interfaces. Universal Access in Human-Computer Interaction. Ambient Interaction. UAHCI 2007. Lecture Notes in Computer Science, vol 4555.

[11] Wichert R, Furfari F, Kung A, Tazari M, Wichert R, Eberhardt B (2012). How to overcome the market entrance barrierachieve the market breakthrough in AAL. Ambient Assisted Living. Advanced Technologies and Societal Change.

[12] Fitzpatrick G, Huldtgren A, Malmborg L, Harley D, Ijsselsteijn W, Wulf V, Schmidt K, Randall D (2015). Design for agency, adaptivity and reciprocity: reimagining AAL and telecare agendas. Designing Socially Embedded Technologies in the Real-World. Computer Supported Cooperative Work.

[13] van Heek J, Himmel S, Ziefle M (2017). Helpful but spooky? Acceptance of AAL-systems contrasting user groups with focus on disabilities and care needs. http://www.scitepress.org/PublicationsDetail.aspx?ID=kUZNUnx8szU=&t=1.

[14] Eberhardt B, Fachinger U, Henke KD (2010). Better health and ambient assisted living (AAL) from a global, regional and local economic perspective. IJBHR.

[15] Randell R, Wilson S, Fitzpatrick G (2010). Editorial — Evaluating New Interactions in Health Care: Challenges and Approaches. Int J Human Comput Interact.

[16] Nunes F, Fitzpatrick G (2015). Self-Care Technologies and Collaboration. Int J Human Comput Interact.

[17] Stone EE, Skubic M (2015). Fall detection in homes of older adults using the Microsoft Kinect. IEEE J Biomed Health Inform.

[18] Ni B, Nguyen CD, Moulin P (2012). RGBD-camera based get-up event detection for hospital fall prevention.

[19] Dohr A, Modre-Opsrian R, Drobics M, Hayn D, Schreier G (2010). The internet of things for ambient assisted living.

[20] Hristova A, Bernardos AM, Casar JR (2008). Context-aware services for ambient assisted living: A case-study.

[21] Costa R, Novais P, Costa A, Neves J (2009). Memory support in ambient assisted living.

[22] Patel S, Park H, Bonato P, Chan L, Rodgers M (2012). A review of wearable sensors and systems with application in rehabilitation. J Neuro Engineering Rehabil.

[23] Isern D, Sánchez D, Moreno A (2010). Agents applied in health care: A review. Int J Med Inform.

[24] Gövercin M, Meyer S, Schellenbach M, Steinhagen-Thiessen E, Weiss B, Haesner M (2016). SmartSenior@home: Acceptance of an integrated ambient assisted living system. Results of a clinical field trial in 35 households. Inform Health Soc Care.

[25] Sun H, De Florio V, Gui N, Blondia C (2010). The missing ones: Key ingredients towards effective ambient assisted living systems. J Ambient Intell Smart Environ.

[26] Wilkowska W, Ziefle M, Himmel S, Tryfonas T, Askoxylakis I (2015). Perceptions of Personal Privacy in Smart Home Technologies: Do User Assessments Vary Depending on the Research Method?. Human Aspects of Information Security, Privacy, and Trust; HAS 2015; Lecture Notes in Computer Science.

[27] van Heek J, Himmel S, Ziefle M, Zhou J, Salvendy G (2017). Privacy, Data Security, and the Acceptance of AAL-Systems – A User-Specific Perspective. Human Aspects of IT for the Aged Population. Aging, Design and User Experience. ITAP 2017. Lecture Notes in Computer Science, vol 10297.

[28] Wireklint Sundström B, Dahlberg K (2011). Caring assessment in the Swedish ambulance services relieves suffering and enables safe decisions. Int Emerg Nurs.

[29] Vicente V, Castren M, Sjöstrand F, Wireklint Sundström B (2013). Elderly patients? Participation in emergency medical services when offered an alternative care pathway. Int J Qual Stud Health Well-being.

[30] Larizza MF, Zukerman I, Bohnert F, Busija L, Bentley SA, Russell RA, Rees G (2014). In-home monitoring of older adults with vision impairment: exploring patients', caregivers' and professionals' views. J Am Med Inform Assoc.

[31] López SA, Corno F, De Russis L (2015). Supporting caregivers in assisted living facilities for persons with disabilities: a user study. Univ Access Inf Soc.

[32] Huryk LA (2010). Factors influencing nurses' attitudes towards healthcare information technology. J Nurs Manag.

[33] Engström M, Lindqvist R, Ljunggren B, Carlsson M (2009). Staff members' perceptions of a ICT support package in dementia care during the process of implementation. J Nurs Manag.

[34] Klack L, Ziefle M, Wilkowska W, Kluge J (2013). Telemedical versus conventional heart patient monitoring: a survey study with German physicians. Int J Technol Assess Health Care.

[35] Davis FD, Bagozzi RP, Warshaw PR (1989). User Acceptance of Computer Technology: A Comparison of Two Theoretical Models. Manage Sci.

[36] Venkatesh V, Morris MG, Davis GB, Davis FD (2003). User Acceptance of Information Technology: Toward a Unified View. MIS Quarterly.

[37] Alagöz F, Ziefle M, Wilkowska W, Calero Valdez A (2011). Openness to accept medical technology - a cultural view.

[38] Beier G (1999). Kontrollüberzeugungen im Umgang mit Technik [Control beliefs in dealing with technology]. Rep Psychol.

[39] Xu H, Dinev T, Smith HJ, Hart P (2009). Examining the formation of individual's privacy concerns: Toward an integrative view. https://aisel.aisnet.org/icis2008/6.

[40] Morton A (2013). Measuring inherent privacy concern and desire for privacy - A pilot survey study of an instrument to measure dispositional privacy concern.

[41] McKnight DH, Choudhury V, Kacmar C (2002). Developing and Validating Trust Measures for e-Commerce: An Integrative Typology. Inform Sys Res.

[42] Himmel S, Ziefle M, Arning K, Kurosu M (2013). From Living Space to Urban Quarter: Acceptance of ICT Monitoring Solutions in an Ageing Society. Human-Computer Interaction. Users and Contexts of Use. HCI 2013. Lecture Notes in Computer Science; Vol. 8006.

[43] Simonazzi A (2008). Care regimes and national employment models. Cambridge J Econ.

