# Supplementary material for: They Don’t Care About Us! Care Personnel’s Perspectives on Ambient Assisted Living Technology Usage: Scenario-Based Survey Study
Source: JMIR Rehabil Assist Technol. 2018 Sep 24;5(2):e10424. doi: 10.2196/10424 (PMC6231777; doi:10.2196/10424)
Supplement: Multimedia Appendix 1 [file rehab_v5i2e10424_app1.pdf]

Multimedia Appnedix 1. Descriptive statistics (means and SDs) for the whole sample and the 3 care contexts.

		Sample (n=170)		Geriatric Care		Nursing Care		Disabled people's Care and Support	
**Construct**	**Items** (evaluation: min=1; max=6)	**M**	***SD***	**M**	**SD**	**M**	**SD**	**M**	**SD**
Attitude toward technical self-efficacy (4 items) **(n.s.)**	I can solve few technical problems alone, that I am faced with.	4.1	1.1	4.5	1.1	3.8	0.9	4.0	1.1
	It's fun to crack a technical problem.	3.4	1.6	3.8	1.6	3.1	1.3	3.2	1.6
	Because I have coped well with previous technical problems, I look optimistically forward to future technical problems.	4.0	1.4	4.6	1.1	3.7	1.2	3.8	1.4
	I feel so helpless about technical problems that I prefer to keep my hands off them.	2.2	1.2	1.9	1.1	2.3	1.1	2.3	1.3
	Index: technical self-efficacy	3.4	0.7	3.7	0.7	3.2	0.6	3.3	0.7
Needs for privacy (6 items) **(n.s.)**	Compared to others, I am more sensitive with regard to handling my data.	3.9	1.2	4.2	1.2	3.6	1.2	4.0	1.2
	Since I have nothing to hide, it does not matter if others know personal information about me.	2.7	1.3	2.8	1.4	2.9	1.1	2.5	1.4
	Compared to others I feel it is more important to keep personal information for me.	4.2	1.2	4.4	1.3	4.0	1.1	4.3	1.1
	Compared to others, I am less worried about my privacy.	2.8	1.2	2.8	1.3	2.8	1.0	2.7	1.1
	I have no problem giving information about me to others as long as they give me no reason not to do it anymore.	3.2	1.3	3.0	1.3	3.4	1.1	3.2	1.4
	I have no problem communicating personal information to other people, including strangers.	2.2	1.1	2.4	1.3	2.4	1.1	2.0	1.0
	Index: privacy	4.2	0.9	4.3	0.9	4.0	0.8	4.3	0.9
Inter-personal trust (3 items) **(n.s.)**	In general, most people keep their promises.	3.7	0.9	3.5	1.2	3.7	0.9	3.7	0.8
	People usually try to follow their words with deeds.	3.6	0.9	3.5	1.0	3.6	0.8	3.6	0.9
	The majority of people are honest in their dealings with others.	3.4	0.9	3.4	1.0	3.5	0.9	3.3	1.0
	Index: interpersonal trust	3.5	0.8	3.5	0.9	3.6	0.7	3.5	0.7
	Perception of Ambient Assisted Living (AAL) technologies								
Types of gathered data[a] *(14 items)*	Care duration (per person)	3.0	1.6	3.1	1.8	3.2	1.3	2.9	1.6
	Times (rooms are entered or left)[a]	2.9	1.4	3.0	1.7	**3.3**	1.2	**2.6**	1.4
	Talks during care	2.1	1.4	2.1	1.8	2.3	1.2	2.1	1.3
	Cries for help/shouts	5.0	1.1	5.0	1.1	5.2	0.9	5.0	1.2
	Whole care situations[a]	2.9	1.6	3.1	1.7	**3.5**	1.4	**2.6**	1.5
	Fixations	4.1	1.6	3.9	1.8	4.5	1.4	4.0	1.6
	Sleeping	3.2	1.5	3.7	1.8	3.0	1.1	3.1	1.5
	Emergency (falls. epileptic seizures)	5.3	0.9	5.5	0.7	5.4	0.7	5.2	1.0
	24h observation[a]	2.6	1.6	**2.9**	1.8	**2.9**	1.5	**2.3**	1.4
	Room data (opening windows. doors...)	4.0	1.6	3.9	1.7	4.4	1.2	4.0	1.6
	Position of caretakers[b]	3.6	1.4	**4.0**	1.5	**4.0**	1.2	**3.2**	1.4
	Position of caregivers	2.7	1.5	2.9	1.7	2.9	1.4	2.6	1.5
	Actuation of emergency button (caretaker)	5.2	1.0	5.3	1.0	5.3	0.8	5.0	1.1
	Actuation of emergency button (caregiver)	5.1	1.0	5.2	0.9	5.4	0.7	5.0	1.1
	Index: types of gathered data	3.7	0.8	3.8	1.0	4.0	0.6	3.5	0.8

Technologies to gather data[b] *(12 items)*	Microphones[a]	2.3	1.4	2.4	1.4	**2.8**	1.3	**2.1**	1.3
	Ultrasonic sensors[b]	3.3	1.5	**3.6**	1.6	**3.8**	1.3	**2.9**	1.5
	Cameras	2.2	1.3	2.4	1.3	2.6	1.4	2.0	1.2
	Infrared cameras[a]	2.5	1.4	2.6	1.5	**2.9**	1.5	**2.3**	1.2
	Motion sensors in rooms[b]	3.4	1.6	**3.8**	1.7	**3.9**	1.2	**2.9**	1.6
	Fall sensors (floor)[b]	4.8	1.4	**5.0**	1.3	**5.2**	0.9	**4.5**	1.5
	Fall sensors (clothing/on the body)[b]	4.3	1.5	**4.7**	1.4	**4.7**	1.2	**3.9**	1.6
	Motion sensors (clothing/on the body of caretakers)[b]	3.3	1.6	**3.9**	1.8	**3.9**	1.3	**2.7**	1.4
	Motion sensors (clothing/on the body of caregivers)[b]	2.5	1.5	**2.9**	1.9	2.7	1.3	**2.1**	1.2
	Room sensors (air, temperature, …)	4.0	1.6	4.3	1.6	4.4	1.3	3.8	1.7
	Emergency button (caretaker)[b]	5.1	1.1	**5.4**	0.7	**5.4**	0.8	**4.8**	1.4
	emergency button (caregiver)	5.1	1.2	5.2	1.2	5.2	0.9	5.0	1.2
	Index: specific AAL technologies	3.6	0.9	3.9	1.0	3.9	0.7	3.2	0.9
Data access (3 items) **(n.s.)**	Data may only be evaluated for the moment.	3.9	1.2	2.7	1.2	3.2	1.2	2.9	1.2
	Data may be stored on a daily basis.	2.9	1.2	2.9	1.3	2.8	1.1	3.0	1.3
	Data may be stored long term.	2.3	1.0	2.5	1.3	2.5	1.0	2.6	1.2
	Index: data access	2.8	1.1	2.7	1.1	2.8	1.0	2.8	1.2
Data storage (3 items) **(n.s.)**	Data may be accessed by colleagues.	2.9	1.2	3.9	1.3	4.0	1.0	3.9	2.8
	Data may be accessed by direct supervisors.	2.9	1.3	2.9	1.3	3.1	1.2	2.8	1.2
	Data may be accessed by all supervisors.	2.5	1.2	2.3	1.0	2.4	1.1	2.2	1.0
	Index: data storage	3.0	0.9	3.1	1.0	3.1	0.7	2.9	0.9
Perceived benefits of AAL technology usage (14 items) **(n.s.)**	Relief in everyday life (caretaker)	3.8	1.3	3.7	1.6	3.9	1.0	3.7	1.3
	Increase in safety (caretaker)	4.2	1.2	4.1	1.3	4.5	0.9	4.2	1.3
	Extension of independence (caretaker)	3.7	1.4	3.8	1.5	4.0	1.2	3.6	1.5
	Decrease of dependence (caretaker)	3.5	1.4	3.4	1.5	3.6	1.1	3.5	1.4
	Relief in everyday life (caregiver)	3.8	1.4	3.8	1.6	3.8	1.2	3.7	1.4
	Fast help in emergencies	4.6	1.2	4.3	1.3	4.8	0.7	4.7	1.2
	Relief in documentation of care (caregiver)	3.9	1.4	3.9	1.5	3.9	1.3	3.9	1.3
	Simplified proof of rendered care (caregiver)	3.9	1.4	3.6	1.6	4.1	1.2	3.9	1.4
	Time savings in everyday working life (caregiver)	3.6	1.3	3.5	1.3	3.8	1.2	3.6	1.2
	Measure against crisis in care (caregiver)	2.8	1.6	2.7	1.6	2.8	1.5	2.9	1.6
	Higher control in everyday working life (caregiver)	4.0	1.5	3.7	1.6	3.8	1.4	4.1	1.6
	Increase of life quality (caretaker)	3.6	1.5	3.4	1.5	3.7	1.2	3.6	1.6
	Increase of working quality (caregiver)	3.5	1.4	3.5	1.6	3.7	1.1	3.5	1.5
	Less fear of being able to do something wrong	2.9	1.5	2.9	1.6	2.8	1.4	3.0	1.4
	Index: perceived benefits	3.7	1.0	3.5	1.1	3.8	0.8	3.7	1.0
Perceived barriers of AAL technology usage (17 items) **(n.s.)**	Confrontation with new technology	3.4	1.4	3.4	1.4	3.3	1.3	3.6	1.5
	Dependence on technology	4.7	1.2	4.4	1.4	4.5	1.2	4.9	1.1
	Surveillance by technology	5.0	1.1	4.9	1.2	4.7	1.3	5.2	1.0
	Invasion of privacy	5.2	1.0	5.1	1.1	5.1	1.0	5.3	1.0
	Recording of data	4.6	1.3	4.6	1.4	4.3	1.3	4.8	1.2
	Transfer of data	4.7	1.3	4.6	1.4	4.4	1.3	4.8	1.2
	Fear of isolation	4.1	1.4	3.9	1.6	3.9	1.3	4.3	1.4
	Substitution of human care by technology	4.8	1.4	4.5	1.5	4.6	1.5	4.9	1.4
	Usage seems to be to complex	3.5	1.3	3.2	1.3	3.3	1.2	3.7	1.4
	Control by colleagues	4.6	1.3	4.6	1.3	4.5	1.4	4.6	1.3
	Control by supervisors	4.9	1.2	4.9	1.2	4.8	1.2	4.9	1.2
	Unauthorized access of data from third parties	4.7	1.2	4.4	1.5	4.5	1.3	4.8	1.1

	Data abuse by third parties	4.8	1.2	4.4	1.3	4.6	1.4	5.0	1.0
	Feeling to be not able to control the technology	3.9	1.4	3.6	1.4	3.5	1.4	4.2	1.4
	Missing trust in functionality of technology	3.9	1.4	3.7	1.5	3.4	1.2	4.2	1.4
	Interruption of routines	3.9	1.3	4.0	1.5	3.7	1.0	3.9	1.3
	Time required for system operation	3.8	1.3	3.4	1.4	3.6	1.2	4.0	1.3
	Index: perceived barriers	4.4	0.7	4.2	0.8	4.2	0.6	4.6	0.7
Acceptance of the described AAL system (6 items)[a]	I can well imagine to use an AAL system.[b]	3.6	1.5	3.8	1.7	**4.1**	1.0	**3.2**	1.5
	An AAL system should be installed in our facility.[a]	3.0	1.5	**3.3**	1.6	**3.4**	1.2	**<u>2.7</u>**	1.4
	I do not want to use such an AAL system.[b]	3.3	1.6	3.0	1.8	**2.8**	1.5	**3.7**	1.6
	I can benefit from an AAL system in many ways.	3.5	1.5	3.8	1.8	3.7	1.1	3.3	1.4
	I do not see any advantage in such a system.	3.1	1.5	3.0	1.7	2.8	1.1	3.3	1.5
	I find the described AAL system useful.[a]	3.6	1.4	3.8	1.7	**3.9**	1.0	**3.3**	1.4
	Index: AAL technology Acceptance[a]	3.5	1.3	3.8	1.6	**3.9**	0.9	**3.3**	1.2

[a]$P<.05$.

[b]$P<.01$.

Means in bold mark the significant group differences based on post-hoc tests (Tukey's honestly significant difference test).
